# Development and validation of a new MRI simulation technique that can reliably estimate optimal in vivo scanning parameters in a glioblastoma murine model

**DOI:** 10.1371/journal.pone.0200611

**Published:** 2018-07-23

**Authors:** Andrea Protti, Kristen L. Jones, Dennis M. Bonal, Lei Qin, Letterio S. Politi, Sasha Kravets, Quang-Dé Nguyen, Annick D. Van den Abbeele

**Affiliations:** 1 Department of Imaging, Lurie Family Imaging Center, Center for Biomedical Imaging in Oncology, Dana-Farber Cancer Institute, Harvard Medical School, Boston, Massachusetts, United States of America; 2 Neuroimaging Research, Radiology Department, Boston Children’s Hospital, Harvard Medical School, Boston, Massachusetts, United States of America; 3 Radiology Department, University of Massachusetts Medical School, Worcester, MA, United States of America; 4 University of Massachusetts Memorial Medical Center, Worcester, MA, United States of America; 5 Department of Biostatistics and Computational Biology, Dana-Farber Cancer Institute, Boston, Massachusetts, United States of America; 6 Department of Radiology, Brigham and Women’s Hospital, Harvard Medical School, Boston, Massachusetts, United States of America; Worcester Polytechnic Institute, UNITED STATES

## Abstract

**Background:**

Magnetic Resonance Imaging (MRI) relies on optimal scanning parameters to achieve maximal signal-to-noise ratio (SNR) and high contrast-to-noise ratio (CNR) between tissues resulting in high quality images. The optimization of such parameters is often laborious, time consuming, and user-dependent, making harmonization of imaging parameters a difficult task. In this report, we aim to develop and validate a computer simulation technique that can reliably provide “*optimal in vivo scanning parameters*” ready to be used for in vivo evaluation of disease models.

**Methods:**

A glioblastoma murine model was investigated using several MRI imaging methods. Such MRI methods underwent a simulated and an in vivo scanning parameter optimization in pre- and post-contrast conditions that involved the investigation of tumor, brain parenchyma and cerebrospinal fluid (CSF) CNR values in addition to the time relaxation values of the related tissues. The CNR tissues information were analyzed and the derived scanning parameters compared in order to validate the simulated methodology as a reliable technique for “*optimal in vivo scanning parameters*” estimation.

**Results:**

The CNRs and the related scanning parameters were better correlated when spin-echo-based sequences were used rather than the gradient-echo-based sequences due to augmented inhomogeneity artifacts affecting the latter methods. “*Optimal in vivo scanning parameters*” were generated successfully by the simulations after initial scanning parameter adjustments that conformed to some of the parameters derived from the in vivo experiment.

**Conclusion:**

Scanning parameter optimization using the computer simulation was shown to be a valid surrogate to the in vivo approach in a glioblastoma murine model yielding in a better delineation and differentiation of the tumor from the contralateral hemisphere. In addition to drastically reducing the time invested in choosing optimal scanning parameters when compared to an in vivo approach, this simulation program could also be used to harmonize MRI acquisition parameters across scanners from different vendors.

## Introduction

A common approach to brain tumor imaging includes anatomical and physiological MRI in order to achieve volumetric and functional evaluations of the disease [1–3]. The anatomical portion of the investigation encompasses the use of pre- and post-contrast MRI spin- and gradient-echo-based sequences. Among these, the Fast-Spin-Echo (FSE) and the Fluid Attenuated Inversion Recovery (FLAIR) as well as the standard Gradient-Echo (GRE) and the Magnetization-Prepared Rapid Gradient-Echo (MP-RAGE) are the preferred MRI methods. These sequences generate high quality images in a limited scan time and provide precise and accurate volumetric measurement of the tissue of interest after appropriate analysis.

Once a tumor is accurately detected and delineated, the MR images can be used to add diagnostic information by examining their T1 and T2 properties. For instance, the signal characteristics of the lesion may provide clues about its underlying composition that are relevant for its characterization [4]. T1 hyperintensity usually denotes fat, subacute hemorrhage, protein-rich fluid, slow vascular flow or contrast enhancement, while T1 hypointensity may represent cerebrospinal fluid (CSF) or a relative increase in tissue water in tumor or areas of edema. T2 hyperintensity associates with relative tissue increase in water such as in edema, tumor cells, inflammation or infection, while T2 hypointensity identifies paramagnetic substances such as deoxyhemoglobin, hemosiderin and iron, calcification and protein-rich fluid. Administration of contrast agent is usually utilized for perfusion and permeability assessments on dynamic scans in addition to highlighting permeable sites on a “static image” acquired several minute after injection. Investigating and optimizing all the MRI aspects underlined in such studies, often necessitates a pre-clinical investigation.

A pre-clinical study of murine models for human cancer requires the identification of mice-bearing tumors and the quantitation of tumor size for stratification, measurements of tumor growth rate, and assessment of treatment response. The optimization of MRI anatomical sequences is often the first step in order to maximize the information available [5]. A murine glioblastoma tumor model was chosen in this study to represent one of the most genetically heterogeneous, resistant and lethal of all human cancers [6]. Several studies in the past have approached the glioblastoma murine model utilizing various in vivo imaging methods [7, 8] with MRI as the methodology of choice whenever a detailed investigation of tumor growth is necessary in a non-invasive manner.

Standardization of MRI scanning parameters is the key for consistent and comparable image quality between examinations in longitudinal and multicenter studies. Although a theoretical approach describing the magnetization evolution during an acquisition is well established, both clinical and pre-clinical studies use scanning parameters that are mostly designed to achieve high quality, but often differ between institutions. In an effort to harmonize the choice of MRI scanning parameters for in vivo examination of a glioblastoma murine model using a 7T scanner, this study aimed at generating reliable computer simulation programs that automatically produce “*optimal in vivo scanning parameters”* as to replace the time-consuming and often inconsistent MRI in vivo parameters optimization approach, while providing a series of guidelines which can be used reproducibly in longitudinal studies and across centers equipped with MRI from different vendors. We investigated several MRI sequences such as FSE, FLAIR, GRE and 3D MP-RAGE in the context of pre- and post- administration of the Gd-DTPA contrast agent, using both an in vivo and simulated MRI of the murine tumor model. The results were then compared and optimal parameters were selected. Such optimal scanning parameters aimed at providing better delineation and differentiation between tissues under study than non-optimized parameters, therefore improving characterization and analysis of the tissue of interest.

The development of a simulated approach and its validation to an in vivo scenario is not a trivial task. In vivo conditions are much more complex than the simulated ones due to resident inhomogeneity and biological conditions. Herein, we demonstrate that achieving “*optimal in vivo scanning parameters”* by simulation is possible and deliverable with the minimal contribution of a set of information achieved in vivo that are targeted to optimize some of the initial simulated scanning parameters.

## Materials and methods

### MRI theory

Theoretical calculation for spin- and gradient-echo-based sequences assume that all radiofrequency (RF) pulses give exactly the desired flip angles, that any effects due to stimulated echoes are not considered, that RF pulses act instantaneously and have the same effect across the whole slice thickness. In addition, it is assumed that all transverse magnetization either decays or is spoiled before each TR (repetition time). The following theory equations are based on a standard Cartesian acquisition and k-space gridding.

### Spin-echo-based sequences

Spin-echo-based acquisitions are used widely in pre-clinical and clinical MRI because they provide a variety of image contrasts that highlights pathology and are resistant to image artifacts from RF and static field inhomogeneity. They can be engaged both as T1- and T2-weighted sequences or to suppress a specific signal such as that of the CSF. The sequences studied in this work are the Fast-Spin-Echo (FSE) and the Fluid Attenuated Inversion Recovery *(*FLAIR).

#### Fast-Spin-Echo (FSE)

Fast-Spin-Echo (FSE) or Turbo-Spin-echo (TSE) pulse sequences are optimized derivatives of the Rapid Acquisition with Relaxation Enhancement (RARE) technique [9]. The primary difference between the more standard method and the FSE lays in the use of a multi-echo approach. FSE combines the desirable properties of spin-echo-based acquisitions with the speed advantage of collecting multiple lines of phase-encoding data following each 90° RF excitation. This method is used for a broad spectrum of MRI applications going from anatomical organ or tumor volume estimation [10] to diffusion imaging [11, 12]. The method can be employed either as a T1- or proton density or a T2-weighted sequence by varying the TR and TE parameters. Short TR (<1000ms) and TE_eff_ (<40ms) were engaged to investigate T1-weighted images while long TR (>4000ms) and TE_eff_ (>40ms) were used for T2-weighted images [13].

The signal intensity general equation presented for a multiple-spin-echo sequence for the n_th_ readout pulse can be reported as follow:

$$S_{n}\approx M_{n}∙exp(-\frac{{TE}_{n}}{T_{2}});$$ (1)

where “n” identifies the n_th_ read out pulse, M_n_ the n_th_ magnetization, TE_n_ is n_th_ echo where T2 is the transverse relaxation time. The magnetization can be expanded as Conturo et al. [14] described after a first-order approximation assuming that the echo spacing between successive echoes (ESP) is << T1:

$$M_{n}{\approx M}_{0}\;\left\{1-exp\;(-\frac{{TD}_{n}}{T_{1}})\right\};$$ (2)

where T_D nth_ is the time interval between the last echo train and the TR period, which is given by:

$$TD_{n}=TR–ETL∙ESP;$$ (3)

the echo train length (ETL) also determines the speed of the FSE sequence. Note that the signal from each echo is also a function of the proton density and the instrumental gain that are intrinsic variables usually not reported in the general signal equation.

#### Fluid attenuated inversion recovery (FLAIR)

T2-weighted fluid attenuated inversion recovery (FLAIR) became a standard and robust approach in clinical magnetic fields strengths for neuroimaging investigation [15, 16]. Therefore the use of this sequence in pre-clinical studies could be especially useful in translational and co-clinical trial designs [17].

FLAIR pulse sequence relies on the application of a single 180° inversion pulse to null the signal from tissues such as the CSF. FLAIR uses a FSE readout where the signal from each echo is a function of the relaxation time TI, T1, T2 decay, as well as the number of ETL, the TR and ESP.

$$M_{n}{\approx M}_{0}\;\left\{1-2exp\;(-\frac{TI}{T1})+exp\;(-\frac{TR-(ETL*ESP)}{T1})\right\};$$ (4)

The equation and its approximations are reported in more details in the manuscript by Meara S. J. P. and Barker G. J. manuscript [10].

### Gradient-echo-based sequences

Gradient-echo techniques have numerous applications both clinically and pre-clinically, such as high resolution anatomic imaging, contrast-enhanced imaging, angiography and perfusion [12, 18–20]. In this study, we simulated a standard gradient-echo technique and a 3D MP-RAGE that are commonly used in the clinic and can be accurately translated to a pre-clinical environment.

#### Gradient-echo (GRE)

Gradient-echo sequence can be used as a T2*- or T1-weighted, rarely as proton density PD sequence by modification of the TR, TE and flip angle parameters.

The signal intensity general equation presented for a gradient-echo-based sequence for the n_th_ readout pulse can be reported as follow:

$$S_{n}\approx M_{n}sin(\theta )∙exp(-\frac{{TE}_{n}}{T_{2}^{*}});$$ (5)

$$M_{n}=\frac{1-exp(-\frac{TR}{T_{1}})}{1-cos(\theta )∙exp(-\frac{TR}{T_{1}})};$$ (6)

where TR is the repetition time between two consecutive RF pulses and *θ* is the flip angle. Note that the signal from each echo is also a function of the proton density and the instrumental gain that are intrinsic variables usually not reported in the general signal equation.

#### Magnetization-prepared rapid gradient-echo (MP-RAGE)

First introduced by Muger and Brookeman [21], the sequence combines the power of magnetization-prepared imaging and rapid 3D gradient echo acquisition techniques to provide excellent T1 tissue contrasts and high spatial resolution images using a short scanning time. Also, MP-RAGE three-dimensional Fourier transform gradient echo acquisition method offers easy reconstruction of any plane and three-dimensional surface contour rendering with cut away post-processing [22]. Although mainly utilized in brain imaging [23], the method can be applied to a vast number of applications.

MP-RAGE signal equation is a function of the time interval between the inversion recovery pulse (TI), T1 and T2* decay, as well as flip angle (*θ*), TR and TE. Signal intensity from the n_th_ read-out pulse is given by [22, 24]:

$$M_{n}=M_{0}\;\left\{\frac{(1-\delta )∙\left(1-\mu^{n-1}\right)}{1-\mu }+\mu^{n-1}∙\left(1-\gamma \right)-\gamma ∙\mu^{i-1}∙\frac{M_{eq}}{M_{0}}\right\};$$ (7)

where:

$$\delta =exp\;\left(-\frac{ESP}{T_{1}}\right);$$ (8)

μ=δ∙cos(θ); (9)

$$\gamma =exp\;\left(-\frac{TI}{T_{1}}\right);$$ (10)

$$\varphi =exp\;\left(-\frac{TD}{T_{1}}\right);$$ (11)

TR=TI+N∙ESP+TD; (12)

where N is the total number of readout RF pulses and M_eq_ is the steady state magnetization after several TRs.

$$\frac{M_{eq}}{M_{0}}=\frac{1-\varphi +\frac{\varphi ∙\cos\left(\theta )∙(1-\delta )∙(1-\mu^{N-1}\right)}{1-\mu }+\varphi ∙\cos\left(\theta )∙\mu^{N-1}-\rho ∙{cos}^{N}(\theta \right)}{1+\rho ∙{cos}^{N}(\theta )};$$ (13)

$$\rho =exp\;\left(-\frac{TR}{T_{1}}\right);$$ (14)

### Contrast to noise ratio (CNR)

The contrast to noise ratio (CNR) between two regions of an image, is defined as the difference in SNR of those regions:

CNR=SNR1–SNR2; (15)

the simulated SNR was calculated as:

$$SNR\approx \frac{{FOV}_{x}∙{FOV}_{y}∙\Delta z∙F_{sequence}\sqrt{NSA}}{\sqrt{BW∙N_{FE}∙N_{PE}}};$$ (16)

where FOV is the field of view, Δz is the slice thickness, F_sequence_ is the signal intensity equation, NSA the number of averages, BW is the total receiver bandwidth and N_FE_ and N_PE_ are the number of frequency encoding and phase encoding, respectively.

Note that the simulated CNR will be only proportional to the contrast to noise obtained in an in vivo experiment due to the simplified nature of the equation that does not consider the electronic impedance of the transmitter/receiver coil or the electric noise produced by our hardware.

### Experiments

#### Validating the simulation approach versus the in vivo approach

The validation of our MRI simulation approach versus the in vivo approach is based on the study of the magnetization effects that each scanning parameter produces when dynamically changed. Only by understanding such effects, will the computer model be able to accurately provide those optimal scanning parameters which, when used in an in vivo experiment, culminate in maximum CNR between tissues of interest.

The in vivo and simulated approaches are introduced below.

#### The in vivo approach

**Fig 1:**
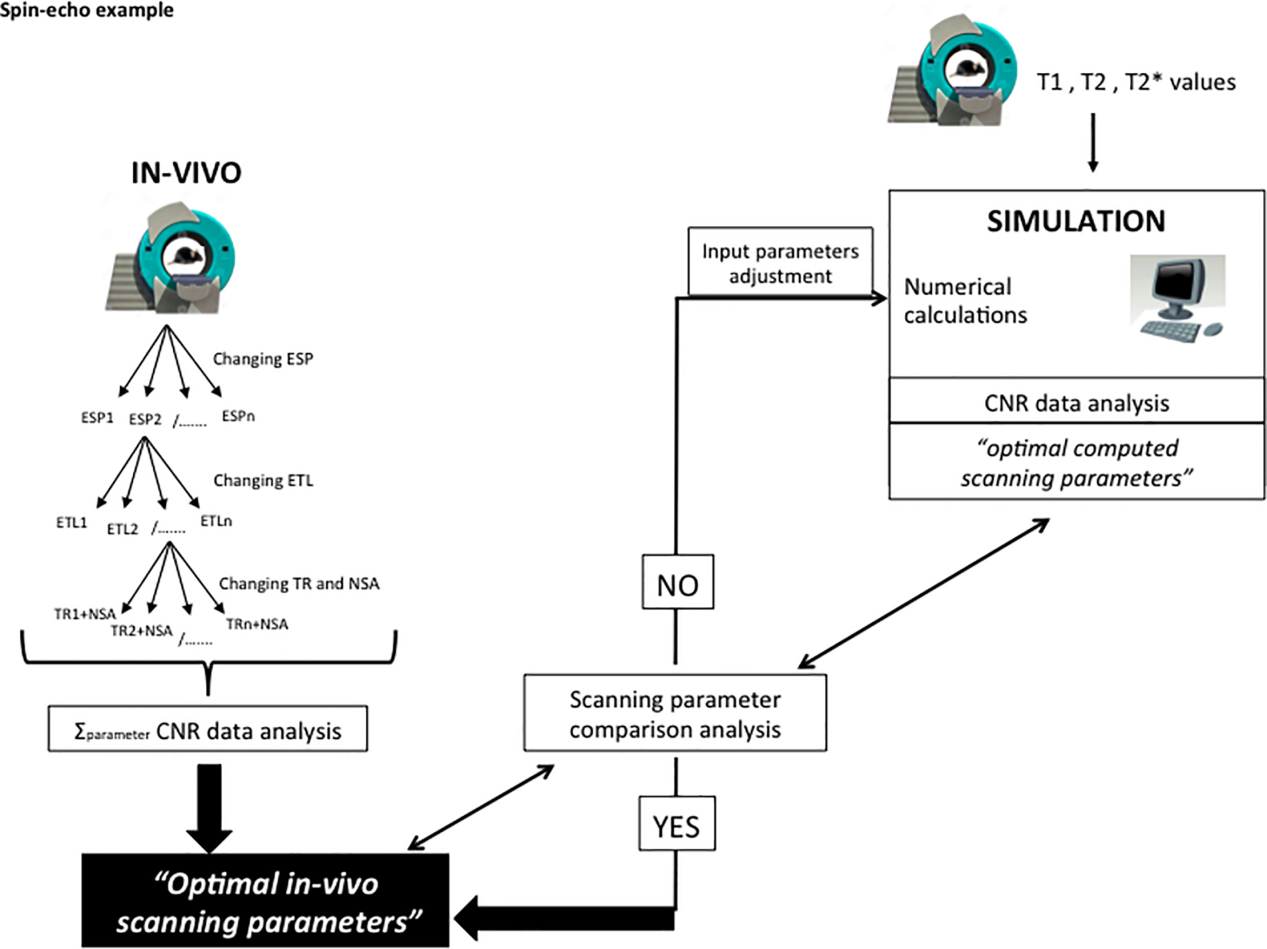
In vivo and simulation approaches diagrams representation. The IN VIVO approach uses a long series of imaging experiments to investigate how the change in MRI scanning parameter affects the SNR of tissues. Once completed, the CNR between selected tissues is analyzed (CNR data analysis) and “*optimal in vivo scanning parameters*” are identified. Conversely, the SIMULATION approach uses a numerical calculation to estimate the magnetization effects on tissues. The simulation uses T1, T2 and T2* values of such tissues, and, in addition, dynamically varies the scanning parameters as input variables. The CNR between selected tissues are then computed and analyzed by the simulation program. “*Optimal computed scanning parameters*” are therefore provided to the user. A verification of the computed scanning parameters versus the in vivo scanning parameters is necessary at this stage (scanning parameter compared analysis). If the outcome is positive (YES), computed and in vivo scanning parameters are similar and the parameter will therefore be considered as an “*optimal in vivo scanning parameter*”. In a negative case (NO), those parameters that differ from the in vivo case, will be reinserted in the simulation emulating the in vivo value and the simulation rerun. Such diagrams are valid in both pre- and post-contrast conditions. Note that the in vivo method is much more time consuming than the simulated approach due to the lengthy parameters’ acquisition experiments that will be mostly replaced by a numerical calculation when using the simulation.

The in vivo approach illustrated on the left side of Fig 1 shows the main steps required to achieve the “*optimal in vivo scanning parameters*”. The method involves a lengthy series of MRI acquisitions as a first step where parameters such as TR, ESP, ETL, flip angle (FA), TI (inversion time) are changed. CNR data analysis between tissues of interest are then performed, maximum CNR values calculated and the related “*optimal in vivo scanning parameters*” found. Of note, in practical terms the in vivo approach cannot be entirely fulfilled because the total scan time is typically constrained to a few hours depending on the diseased animal model in use. However, restricting the scanning conditions to a limited number of parameters, still allows the “*optimal in vivo scanning parameters*” to be accurately defined.

The in vivo approach with limited parameters was engaged in FSE, GRE and MP-RAGE for T1- and T2-weighted acquisitions both in pre- and post-contrast conditions. FLAIR was run only as a T2-weighted pre-contrast.

#### The simulation approach

Our MRI computer simulation (C++ code) computed all the equations presented in the *Theory* section to determine the SNR of brain parenchyma, CSF and tumor tissues and the related CNR. The computer simulation varied, in a dynamic fashion through a numerical calculation, parameters such as ESP, ETL, TR, NSA, FA, TI and scan time in order to study the effects of the latters on the magnetization. The range, within which the parameters were studied, was similar between in vivo and simulation, in fact, the latter mirrored all the in vivo experiments. In a second step, the simulation computed the SNRs of the tissues under study and produced a CNR analysis to estimate the maximum values. Such values exposed the “*optimal computed scanning parameters*”. In order to associate the latter computed parameters to the “*optimal in vivo scanning parameters*”, a scanning parameter comparison analysis was performed between optimal in vivo and computed scanning parameters. In those cases where the comparison analysis failed, the initial simulated conditions (input variables) had to be adjusted to the in vivo optimum value and the simulation repeated (right side of Fig 1).

#### Cell line

The luciferase-transduced murine glioblastoma cell line GL261-luc2 was purchased (Perkin-Elmer) in 2014 and chosen for its syngeneic ability in the murine model. Cells were cultured and expanded in DMEM supplied with 10% FCS and selected under 100μg/mL G418. Cells were grown on culture flasks housed in an incubator maintained at 5% CO_2_ set at 37°.

#### Murine model

All procedures and imaging protocols for this study were approved and performed in accordance with Dana-Farber Cancer Institute’s Institutional Animal Care and Use Committee (IACUC). For the intercranial injection of the GL261-luc2 cells, eight 6–10-week old female B6(Cg)-Tyr^c-2J^/J mice (The Jackson Laboratory, Bar Harbor, ME) were anesthetized for the duration of the procedure using 2% isoflurane mixed with medical air, and placed on a stereotactic frame. The skull of the mouse was exposed through a small skin incision, and a burr hole was made using a 25G needle at 2.0 mm lateral of the bregma. The GL261-luc2 cells (1 x 10^5^ cells in 2μL PBS) were then loaded into a 33G Hamilton syringe and injected 2 mm below the cortical surface of the brain over a one-minute time span.

After suturing the scalp, mice were given a topical anesthetic (Bupivacaine) and an intraperitoneally injected (IP) analgesic (0.05mg/kg buprenorphine). They were then returned to their cages, placed on a warming pad and visually monitored until full recovery. Mice were checked daily for any signs of distress, which included weight loss, dehydration, neurological symptoms such as tremors, seizures, ataxia, and any skull deformation due to advanced tumor growth. Dependent upon the severity of any observed symptoms, body condition scoring and/or tumor size (>500 mm^3^ calculated from the analysis of MR images), the mice were euthanized by CO_2_ asphyxiation in accordance with DFCI IACUC protocols. No animals died due to the experimental procedures. The median survival of the tumor-bearing mice without any therapeutic intervention was approximately 28 days post-tumor cell injection.

#### MRI imaging

MRI in vivo studies were performed on 8 glioblastoma-bearing mice at about 20 days post-cell injection using a 7T/30 cm USR horizontal bore Superconducting Magnet System 300.3 MHz (Bruker BioSpin MRI, Ettlingen, Germany BioSpec). The scanner was equipped with the B-GA12S2 gradient and integrated with up to 2^nd^ order shim system, which provides a maximum gradient amplitude of 440 mT/m and slew rate of 3440 T/m/s. The Bruker-made transmit/receiver 23 mm ID birdcage volume radiofrequency (RF) coil was used for brain images.

Anesthesia was maintained at a flow rate of 2 l/min through inhalation of a mixture of 1.5% isoflurane and O_2_. Body temperature was maintained at 37° using a forced warm air fan system. Animal respiration and temperature were monitored and regulated by the SAII (Stony Brook, NY) monitoring and gating system model 1025T. The mice were injected intraperitoneally (IP) with 0.5 mmol/kg of gadopentetate dimeglumine Gd-DTPA (Magnevist, Bayer, Germany). Pre-contrast acquisitions were obtained in addition to post-contrast images. Post-contrast imaging started at 30 min post-injection.

Bruker Paravision 6.0.1 was used for MRI data acquisition. The geometry and pixel size were maintained constant throughout all the sequences. To limit inhomogeneity correction typical of regridding techniques, in addition to minimizing acquisition scheme artifacts [25, 26], a Cartesian acquisition scheme with no undersampling was preferred to non-Cartesian. For the 2D brain images, a FOV = 20x20 mm^2^, matrix size = 192x192x29 and slice thickness = 0.5 mm were used, while a FOV = 20x20x20 mm^3^ and a matrix size = 128x128x128 were used for the 3D acquisition. The sequences used in this study were FSE and FLAIR as spin-echo based sequences and GRE and MP-RAGE as gradient-echo based sequences.

The scanning parameters used for the parameters’ optimization of the in vivo portion of the study were chosen within standard ranges (typically utilized in preclinical mice experiments) in order to render the study more effective and less time-consuming. Details of such parameters are discussed in the following sections.

#### T1, T2 and T2*

To estimate T1, T2 and T2* values of brain parenchyma, CSF and tumor areas, a spin-echo sequence with variable TR and TE (RAREVTR) was used for simultaneous T1 and T2 mapping. Scanning parameters were: 7 T1 experiments TR = 7000, 5500, 3000, 1500, 800, 400, 278 ms, 5 T2 experiments ESP = 8.5, 25.5, 42.5, 59.5, 76.5 ms, NSA = 1, FA = 90/180, ETL = 2, BW = 50 MHz, matrix = 128x128x3, FOV = 20x20x3 mm^3^, scan time ≈ 15 min. T2* mapping was achieved with the use of a multi-echo GRE sequence as follows: TR = 1000 ms NSA = 2, TE = 2.4 (BW = 75 MHz), echoes = 15, ESP = 4ms, FA = 30; matrix = 128x128x3, FOV = 20x20x3 mm^3^, scan time = 4 min 16 sec.

The sequences were run on all 8 glioblastoma brain tumor-bearing mice. Notably, attainment of T1 and T2 spin echo contrast between white and gray matter is generally more challenging at high B0 rather than low magnetic fields, thus implying a convergence in relaxation values between such tissues at high fields [27]. For this reason, the relaxation times achieved in the contralateral hemisphere were, most probably, a combination of the two relaxation tissues.

#### Fast-Spin-Echo (FSE)

The glioblastoma brain tumor murine model was investigated with a Fast-Spin-Echo in both T2 and T1-weighted pre- and post-contrast. In T2-weighted (FSET2) examinations, the first step was to evaluate the magnetization effects while varying ESP and maintaining the other parameters constant. The following initial parameters were used: TR = 6000 ms, NSA = 1, ETL = 13, ESP changed from 5.4 to 6, 7.4, 9.4, 11.4, 13.4, 15.4, 17.4 ms with consequent BW variation (from 100 to 13.5 MHz). After CNR data analysis, the best ESP for in vivo studies was determined. In a second step, the experiment was repeated maintaining ESP to the founded value but varying ETL from 10 to 20 (increment of 1). The last step was to change the TR from 4000 to 10000 ms in 1000 ms increments while engaging the maximum number of averages (NSA) without exceeding the 5-minute scanning limit. These steps determined the “*optimal in vivo scanning parameters*”.

The Fast-Spin-Echo T1-weighted (FSET1) sequence was performed following similar steps and scan time restrictions of 5-minute acquisitions. Images parameters were initially set to TR = 700 ms, NSA = 3, ETL = 1 and ESP varied from 5.4 to 6, 7.4, 9.4, 11.4, 13.4 ms (BW from 100 to 20 MHz) in the first step. Having found the optimal ESP, ETL was then changed from 1 to 5. Maintaining ESP and ETL at their best value, TR was then changed from 500 (minimum TR) to 800 ms to investigate TR contribution on the SNR of normal and tumor tissues, while using the maximum number of NSA at the same time without exceeding the 5-minute scanning limit. “*Optimal in vivo scanning parameters*” were then determined.

Of note, FSET2 ETL values lower than 10 were not utilized because of scan time limits, while using ETL higher than 20 would have encountered significant SNR drops, therefore deteriorating image quality. Also, the recovery time in the FSET2 method did not fall below 4000 ms in order to maintain strong T2-weighted effects. Similarly, in the FSET1 method, TR did not overcome 1000 ms to maintain T1-weighted effects.

#### Fluid attenuated inversion recovery (FLAIR)

In the FLAIR pre-contrast method, the following initial parameters were used: TR = 8000 ms, ESP = 7.4 (BW = 50 MHz), ETL = 1, NSA = 1 and TI varied from 1100 to 3100 ms (200 ms increment). Such experiment meant to assess the best TI value for the in vivo applications. In a second step, TI was maintained at the optimal in vivo value while TR ranged from 6000 to 25000 ms (non-constant increments). In this test, the scanning time of each experiment was limited to 10 minutes, and as a consequence NSA was maintained equal to 1, while the ETL number was limited to the minimum value (TR = 6000 ms ETL = 2; TR = 7000 to 9000 ETL = 3; TR = 10000 to 11000 ms ETL = 4; TR = 15000 ms ETL = 5; TR = 20000 ms ETL = 7; TR = 25000 ms ETL = 9). This step provided optimal TR and ETL to complete the “*optimal in vivo scanning parameters*”.

#### Gradient-echo (GRE)

The GRE sequence was used as a T1-weighted pre- and post-contrast method on the tumor-bearing mice. As a pre-contrast sequence, it offered low CNR between the brain parenchyma and tumor tissues while producing good contrast between soft tissue, blood and CSF. As a first step, GRE parameters were set to TR = 275 ms (minTR), TE = 3.5 (BW = 50 MHz), NSA = 3, variable FA (from 10 to 90 degrees) in order to investigate FA effects on the magnetization. In a second step, the FA was maintained to the optimal value and TR varied from 275 to 350, 500, 700 and 1000 ms combined with the maximum number of NSA without exceeding the 5-minute scan time limit. “*Optimal in vivo scanning parameters*” were then defined.

#### Magnetization-prepared rapid gradient-echo (MP-RAGE)

MP-RAGE examination was engaged as a T1-weighted pre- and post-contrast agent method. To assess the optimal TI parameters for the in vivo study of the glioblastoma murine model, the sequence parameters were set to: TR = 1983 (minTR), TE = 2.3 ms (minTE) (BW = 75 MHz), segment = 1, NSA = 1, FA = 10°, and TI varied between 500 and 3500 ms (500 ms increment). In a second step, while maintaining the TI constant at the optimal in vivo value, the FA was changed from 10° to 90° and NSA was maximized in a scan time limit of 5 minutes. The analysis of CNR values provided the “*optimal in vivo scanning parameters*”.

### Data analysis

T1, T2 and T2* values were calculated by employing the Image Sequence Analysis (ISA) Tool provided by Bruker Paravision which uses dedicated mathematical functions to best fit the data. The same region of interest (ROI) were maintained throughout the dataset for the analysis of brain parenchyma, CSF and tumor tissues. Tumor ROIs were placed in 3 consecutive slices, typically in the middle of the tumor, to achieve a T1, T2 and T2* value representative of the entire tumor volume. T1, T2 and T2* mean values ± standard deviations (SD) were calculated for individual mice as well as for the entire cohort by averaging over the three slices. The Wilcoxon signed rank test was used to compare T1, T2, and T2* values averaged over three slices from brain parenchyma, CSF, and tumor tissues before and after contrast administration. To assess the variability in T1, T2 and T2* values in brain parenchyma, CSF, and tumor tissues, a repeated measures Analysis of Variance (ANOVA) model was fit for each T1, T2, and T2* variable. Mice were included as a fixed effect to evaluate the differences in values between the mice, and a random effect with slice measurements nested within each mouse was included to evaluate the variability within each mouse.

The SNR values from the in vivo data were analyzed with the ImageJ software (National Institutes of Health, Bethesda, MD) by placing ROIs in the contralateral hemisphere, in CSF and in tumor areas. The analysis was typically repeated in 3 consecutive slices and a CNR mean value ± SD in addition to the coefficients of variation were also derived. A CNR data analysis specific to each scanning parameter was then performed and the highest CNR values assessed. In order for a scanning parameter to be evaluated as an “*optimal in vivo scanning parameter*”, two conditions had to be satisfied: 1) the image had to be free of artifacts derived either from off resonance effect, motion, chemical shift or blurring effects, therefore assuring good image quality (image quality analysis test); 2) the CNR had to be found maximized. CNR mean values ± SD and the related coefficients of variation were calculated for individual mice as well as for the entire cohort.

In the simulation case, the data analysis of the computed CNR values was integrated in the computer program. Due to the absence of an image quality analysis test, the highest computed CNR values always identified the “*optimal computed scanning parameters*”. Such scanning parameters were then compared to the optimal scanning parameters derived by the in vivo study as part of the “scanning parameter comparison analysis”. When the simulated matched the in vivo parameters, the “*optimal computed scanning parameters*” could be considered as “*optimal in vivo scanning parameters*” thus validating the simulation.

To assess the similarity between in-vivo and simulated CNR data, we computed the correlation of CNR data from the two different methods using Kendall's tau coefficient (τ). The coefficient ranges from -1 to 1, with values closer to -1 or 1 indicating higher negative (-1) or positive (1) correlation.

## Results

### T1, T2 and T2* values

Table 1 reports the mean value ± SD for T1, T2 and T2* values at 7T for brain parenchyma, CSF and tumor tissues in the 8 tumor-bearing mice. We find that T1, T2, and T2* values did not change significantly from pre- to post-contrast acquisitions in non-diseased brain parenchyma and CSF. In tumors tissues, while T2 did not change significantly from pre- to post-contrast acquisitions, a significant decrease was observed in T1, as expected, and T2* values in tumors from pre- to post-contrast acquisitions (p-value = 0.004 and 0.022, respectively). The variability within mice was generally smaller than the variability between mice (S1 Table). The latter variability between mice resulted in all cases smaller or equal than 10% of the corresponded mean value. The similar T1 values found before and after contrast administration in both CSF and non-diseased brain parenchyma could be explained by the integrity of the blood brain barrier in healthy brain tissues, in addition to the rapid washout of the contrast agent.

**Table 1:** T1, T2 and T2* values (mean ± SD in the 8 tumor-bearing mice) for brain parenchyma, CSF and tumor tissues of a glioblastoma murine model under pre-contrast and post-contrast conditions.

	Pre-contrast			Post-contrast (30 minutes)		
	Brain parenchyma	CSF	Tumor	Brain parenchyma	CSF	Tumor
**T1±SD (ms)**	2565.13±161.81	3553.53±125.49	3221.08±118.61	2491.88±76.09	3477.46±137.74	1032.96±49.19
**T2±SD (ms)**	46.04±1.02	165.75±16.20	68.67±2.15	47.38±2.07	154.08±7.06	63.79±5.47
T2*±SD (ms)	27.46±1.60	91.91±25.20	37.71±1.86	26.29±0.86	82.88±15.51	34.33±0.99

### Spin-echo based sequences

**Fig 2:**
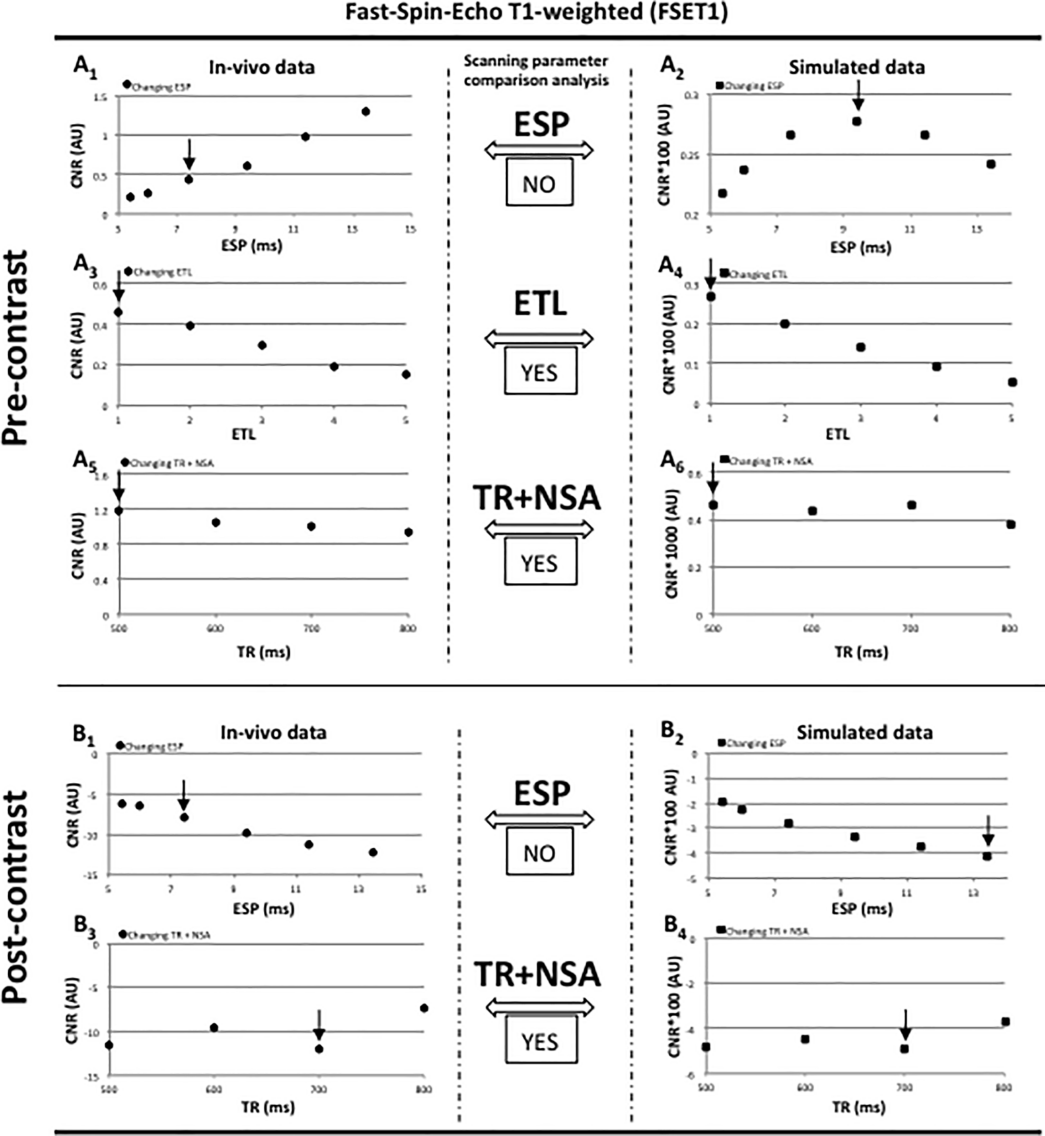
Fast-Spin-Echo CNR data and scanning parameters comparison analysis. Fast-spin-echo T1-weighted (FSET1) brain parenchyma-tumor mean CNR graphical representation. In pre-contrast conditions: graphs A_1,3,5_) and A_2,4_,_6_) report respectively in vivo and simulated CNR FSET1 data when changing ESP, ETL, TR and NSA (the scan time was limited to 5 minutes). In post-contrast conditions: graphs B_1_,_3_) and B_2_,_4_) reported respectively in vivo and simulated CNR FSET1 data when changing ESP, TR and NSA (the scan time was limited to 5 minutes). Black arrows point at selected parameters that typically coincide with the highest CNR providing the *“optimal in vivo scanning parameters”* for the in vivo method, and the “*optimal computed scanning parameters*” with the simulated approach. The latter parameters are compared by the “Scanning parameters comparison analysis” and the outcome reported as successful (YES) or failing (NO). Note that although the graphs are related to a single mouse dataset, they are a good representation of the entire animal cohort.

The results for the procedures of parameter optimization (pre- and post-contrast conditions) are presented in Fig 2 for FSET1 and in S1 Fig for all the spin-echo based cases. The reported graphs are representative of a single mouse in vivo experiment, but are representative of the entire animal cohort. Mean CNR values for individual animals were associated with a coefficient of variation typically smaller than 10%. A similar outcome was also found when calculating the coefficient of variation of the entire cohort thus supporting the accuracy of our measurements and the repeatability of the mouse model. Error bars are not included in the graphs.

It should to be noted that the magnitude of the computed and in vivo single CNR values differ significantly from each other primarily due to the complexity in simulating a real MRI experiment. In fact, the scanner hardware on one end, and the complex biological tissues composition on the other, are primary factors for such magnitude discrepancies. In the case of a MRI scanner, the electronic and hardware components deeply affect both acquisition and amplification of the MRI signal making it unique to every machine. The software used for the in vivo data analysis can also represent a contributing factor to the final magnitude. Black arrows reported in Fig 2 and S1 Fig identified the optimal parameters found for the in vivo and simulated approach.

A full view of the “*optimal in vivo scanning parameters*” spin-echo base sequences is reported in Table 2.

**Table 2:** Pre- and post-contrast “*optimal in-vivo scanning parameters*” for spin-echo and gradient-echo-based sequences in a glioblastoma murine model.

	Pre-contrast						Post-contrast	
	Spin-Echo based			Gradient-Echo based		Spin-Echo based	Gradient-Echo based	
	FSET2	FSET1	FLAIRT2	GRET1	MP-RAGET1	FSET1	GRET1	MP-RAGET1
**TR (ms)**	7000	500	9000	350	1983	500	275	1983
**ESP (ms)**	7.4	7.4	7.4			7.4		
**TEeff (ms)** in SE-based **TE (ms)** in GE-based	59.2	7.4	14.6	3.5	2.3	7.4	3.5	2.3
**BW (MHz)**	50	50	50	50	75	50	50	75
**ETL**	15	1	3			1		
**FA**	90/180	90/180	180/90/180	60	10	90/180	90	10
**NSA**	3	3	1	4	1	3	5	1
**TI (ms)**			1700		1700			1700
**Matrix**	192x192x29	192x192x29	192x192x29	192x192x29	128x128x128	192x192x29	192x192x29	128x128x128
**FOV (mm)**	20x20x14.5	20x20x14.5	20x20x14.5	20x20x14.5	20x20x20	20x20x14.5	20x20x14.5	20x20x20
**Mode**	2D	2D	2D	2D	3D	2D	2D	3D
**Scan time (min : sec)**	4 : 12	4 : 48	9 : 36	4 : 28	4 : 14	4 : 48	4 : 24	4 : 14

#### Fast-Spin-Echo (FSE)

In-vivo and simulated CNR data were highly correlated for most of the parameters analyzed as in Fig 2 and S1 Fig (Kendall's tau coefficient τ for FSET1 is: 0.36 A1—A_2_; 1 A3 –A_4_; 0.55 A5 –A_6_; 1 B1—B_2_; 1 B3 –B_4_); 0.93 S1A1 –S1A_2_; 0.55 S1A3 –S1A_4_; 0.43 S1A5 –S1A_6_). This was found in pre- and post-contrast conditions for both T2- and T1-weighted dataset, confirming that the FSE simulation is a reliable method in reproducing real case scenarios and thus achieves optimal scanning parameters qualification.

Fig 2A_1_ and 2B_1_ highlighted the optimal ESP in vivo value after assessing CNR in combination with the related image quality as described in the “Data Analysis” section. It was found that when using echo spacing larger than 7.4 ms (50MHz) in the T1-weighted methods, and similarly in the T2-weighted methods, chemical shift and distortion artifacts were introduced resulting in significantly diminished image quality. Hence, using 7.4 ms resulted in a good compromise between in vivo maximal CNR and artifacts’ minimization. On the other hand, the simulation (Fig 2A_2_, 2B_2_) selected the best ESP simply based on the CNR magnitude. The in vivo and simulated ESP values were different, therefore failing the scanning parameter comparison analysis. ESP was then re-inserted in the computer program and maintained constant at the optimal in vivo value. When changing either ETL (Fig 2A_3_) or TR in addition to NSA (Fig 2A_5_ and 2B_3_), the simulation (Fig 2A_4_, 2A_6_ and 2B_4_) computed well the magnetization effects observed in the in vivo experiments, resulting in a successful scanning parameter comparison analysis.

#### Fluid attenuated inversion recovery (FLAIR)

S1C_1_ and S1C_2_ Fig reported a similar data trend when varying TI (high Kendall's tau coefficient, 0.75). Both in vivo and simulation approaches identified the highest CNR value at a TI of 1700 ms. A different outcome was found when changing the TR parameter in addition to ETL (S1C_3_ and S1C_4_ Fig low Kendall's tau coefficient, -0.28). The in vivo CNR values dropped after the 9000 ms in contrast to the simulation where the CNR continued to rise until reaching a maximum at a TR of 20000 ms. The unexpected in vivo trend was thought to be related to the increasing number of ETL used at longer TR than 9000 ms (ETL>3) causing inhomogeneity resulting in dephasing effects, which in turn produced a signal dropping. The selected optimal MRI parameters were, therefore, chosen as 1700 ms TI and 9000 ms TR.

### Gradient-echo based sequences

**Fig 3:**
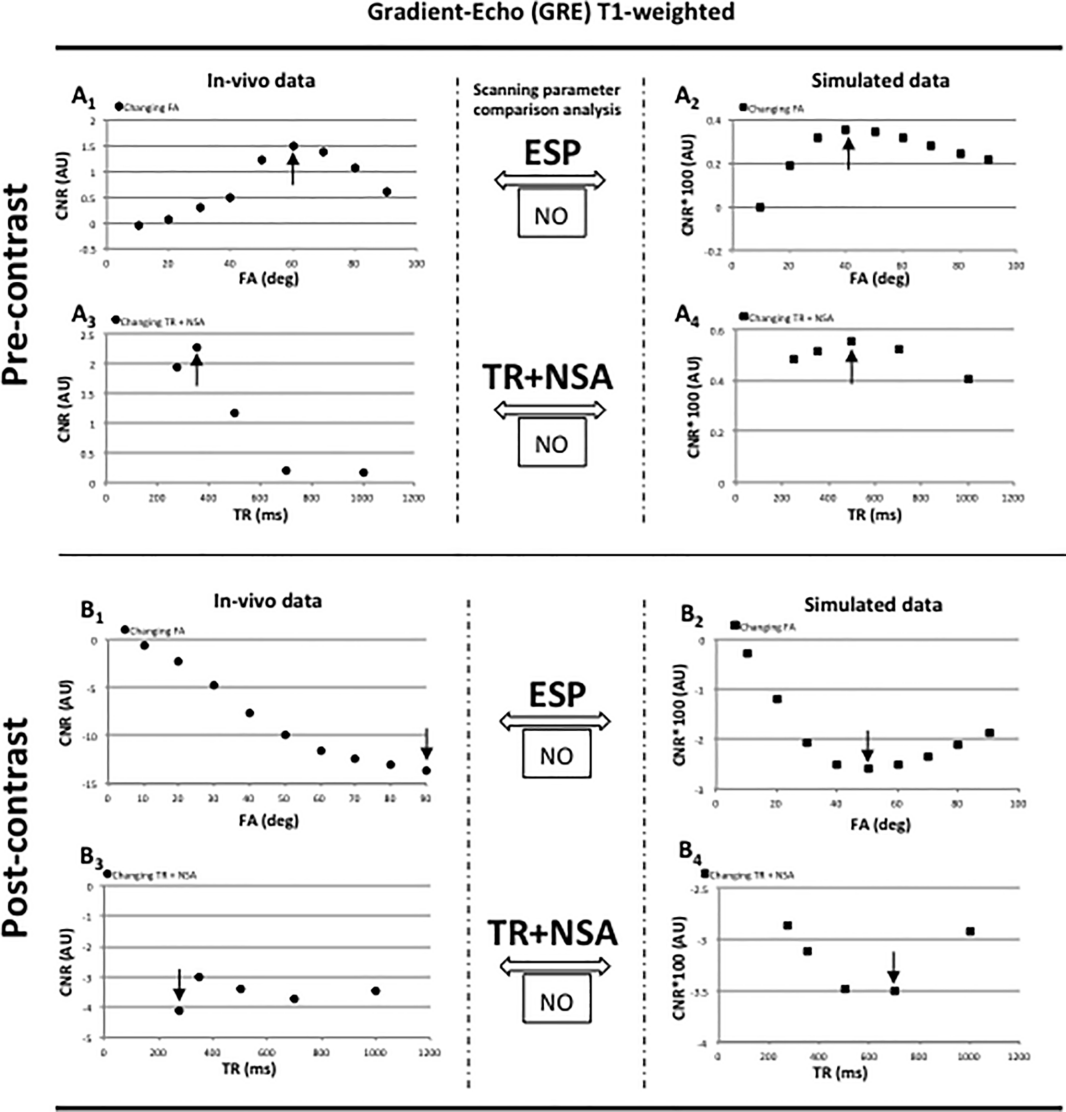
Gradient-echo based sequences diagrams. Gradient-echo (GRE) brain parenchyma-tumor mean CNR graphical representation during scanning parameters optimization. Figs A_1,3_ and A_2,4_ report respectively in vivo and simulated CNR GRE data when changing FA and TR in addition to NSA (5-minute scan limit) in pre-contrast conditions. Same approach is shown in Figs B_1_,_3_ and B_2_,_4_ in post-contrast conditions. Black arrows point at selected parameters that typically coincide with the highest CNR providing the *“optimal in vivo scanning parameters”* in the in vivo approach and the “*optimal computed scanning parameters*” in the simulated approach. The latter parameters are compared with the “Scanning parameters comparison analysis” and the outcome was reported as successful (YES) or failing (NO). Note that although the graphs are related to a single mouse dataset, there is a good representation of the entire animal cohort.

Gradient-echo based sequences results are presented in pre- and post-contrast conditions in Fig 3 and S2 Fig. Although these graphs represent the case of a single mouse, they are a good representation of the entire animal cohort. Mean CNR values were associated to a standard deviation (SD) typically <10%. A coefficient of variation <10% was also found for the entire cohort. Error bars are not included in the graphs. It has to be noted that the magnitude of the computed and in vivo single CNR values differ significantly, primarily due to the complexity in simulating the effect that the scanner hardware has on the acquisition and amplification of the MRI signal. The analysis software for the in vivo data was an additional factor of magnitude values differences. Black arrows reported in Fig 3 and S2 Fig identified the optimal parameters found for the in vivo and simulated approach. The TE was related to a bandwidth of 50MH, as in spin-echo methods, which provided the highest CNR values between tissues based on an in vivo test (data not shown).

A full view of the “*optimal in vivo scanning parameters*” gradient-echo base sequences is reported in Table 2.

#### Gradient-echo (GRE)

The GRE pre- and post-contrast in vivo data trend showed important differences with the simulated counterpart when changing both FA (Fig 3A_1,2_ and 3B_1,2_) and TR in addition to NSA without exceeding a 5-minute scan time limit (Fig 3A_3,4_ and 3B_3,4_). The reasons behind such divergences are not entirely understood, although it can be speculated that enhanced susceptibility effects experienced at high field played a major role. The GRE method is indeed very sensitive to variations in T2* decaying which might have not been fully integrated in the simulation. The optimal parameters were, therefore, chosen based on the highest CNR achieved in the in vivo experiment as 60 degrees angle FA and 350 ms TR in pre-contrast conditions, and 90 degrees angle and 275 ms TR in post-contrast conditions. The correlation between in-vivo and simulated graphics patterns was also low, reporting low Kendall's tau coefficient values (0.39 3A_1_ - 3A_2_; 0.20 3A_3_ - 3A_4_ and 0.22 S2C_1_ –S2C_2_; -0.20 S2C_3_ –S2C_4_)

#### Magnetization-prepared rapid gradient-echo (MP-RAGE)

MP-RAGE reported a non-matching CNR trend (low Kendall's tau coefficient, -0.17) between the in vivo and simulated data in pre-contrast conditions when testing the best TI parameter (S2B_1,2_ Fig). Such differences were also reflected in the failing of the scanning parameter comparison analysis. The reasons behind such dissimilarities remain unclear. In all the remaining cases (S2B_3,4_, S2D_1,2_ and S2D_3,4_ Fig) in vivo and simulation CNR seem to correlate well (high Kendall's tau coefficient 0.80 B3 –B_4_; 0.67 D1 –D_2_; 1 D3 –D_4_) also identifying similar optimum scanning values. Optimal parameters were set as 1700ms TI and 10 degrees FA for both pre- and post-contrast conditions.

### “Optimal in vivo scanning parameters”

The simulation technique wanted to achieve the *“optimal in vivo scanning parameters”* after a validation process that compared the computed scanning parameters to those achieved in vivo. Included is a list of those parameters that succeed and those that failed the scanning parameter comparison analysis.

Successful comparison scanner parameter analysis:

**Table:** 

1) ETL, TR, NSA	in the case of FSE T1 or T2	(pre- and post-contrast);
2) TI	in the case of FLAIR T2	(pre-contrast);
3) FA, NSA	in the case of MP-RAGE T1	(pre-contrast);
4) TI, FA, NSA	in the case of MP-RAGE T1	(post-contrast);

Failed comparison scanner parameter analysis:

**Table:** 

1) ESP	in the case of FSE T1 or T2	(pre- and post-contrast);
2) TR	in the case of FLAIR T2	(pre-contrast);
3) FA, TR, NSA	in the case of GRE T1	(pre- and post-contrast);
4) TI	in the case of MP-RAGE T1	(pre-contrast).

These latter parameters were re-inserted in the simulation and maintained constant at the optimum in vivo experimental value.

The FOV and matrix parameters were not modified throughout the study, and it is believed that their contribution does not affect the series of scanning parameters succeeding or failing the compared analysis.

**Fig 4:**
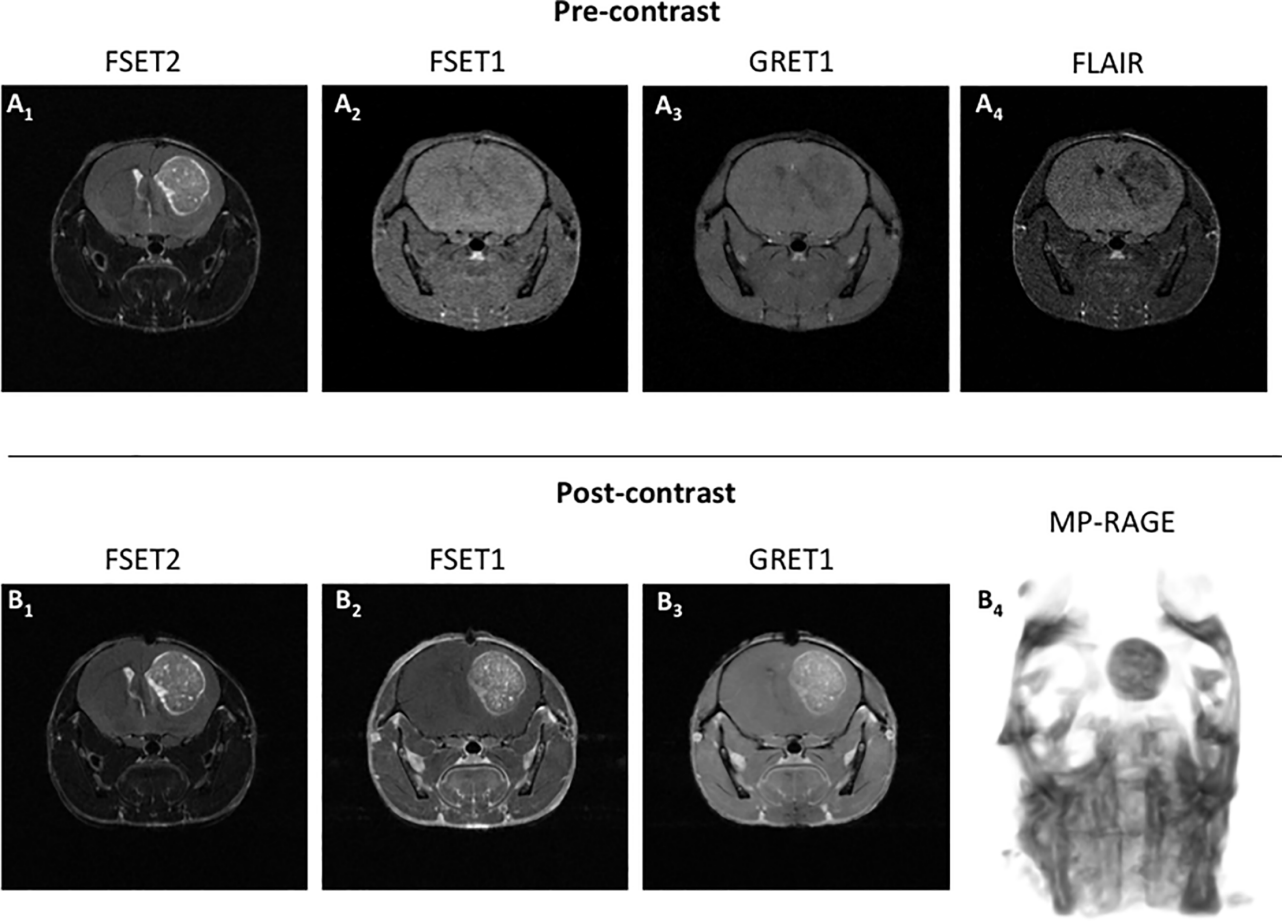
Pre- and post-contrast images “optimal in vivo scanning parameters” of a glioblastoma murine model brain section. Figs A_1_, A_2_, A_3_ and A_4_ represent FSET2 FSET1, GRE and FLAIR pre-contrast images respectively. Tumor areas are well differentiated from the valuable brain parenchyma in FSET2. On FLAIR images, excellent CSF signal saturation is achieved. Figs B_1_, B_2_, B_3_ and B_4_ represent FSET2 FSET1, GRE and MP-RAGE (the image contrast was inverted for visualization purposes) post-contrast images respectively. Tumor areas are well differentiated from the healthy brain parenchyma in all scans. Contrast enhancement effects are prominent in all T1 examinations.

Example of “*optimal in vivo scanning parameters*” resulting images are reported in Fig 4. These images and the related optimal parameters are representative of the entire cohort. This was verified by running the in vivo and simulation approaches on all the animals ultimately finding the same results which was also confirmed by coefficient of variation typically smaller than 10% of the mean value, and the generally small variabilities between and within mice. In other words, if any of the single animal CNR values were to be investigated, the “*optimal in vivo scanning parameters*” outcome would have been identical to that shown herein.

Regarding the time spent running the in vivo compared to the simulated method, it was found that the computed approach provided significant benefits with the exception of the GRE method. The user performing the simulated method will have, in fact, to investigate exclusively the “failed” scanning parameters by an in vivo test and acquire either a T1 or T2 or T2* map depending on the sequence theoretical requirements. Conversely, a full sequential in vivo approach involves acquiring a series of scans for each of the scanning parameters in addition to a full data analysis. For instance in the case of spin-echo, it is estimated that, based on our MRI methods and their acquisition timing, the simulated technique is at least twice as fast as the conventional method in identifying the “*optimal in vivo scanning parameters*”.

## Discussion

This study aimed to validate a numerical simulation based on MRI theory to an in vivo approach in a glioblastoma murine model in order to provide “*optimal in vivo scanning parameters*”. Such parameters produce the highest CNR between healthy and tumor brain tissues which typically translate to an increase in sensitivity and accuracy of the subsequent image analysis. The computational approach simulated similar sequences to those used in an in vivo experiment in an effort to reproduce the magnetization effects observed in a living mouse. The SNR of healthy and tumor tissues collected with both in vivo and simulated approaches were then analyzed through a CNR data analysis, and the respective scanning parameters were compared. While it was found that the computational methods suffered several inaccuracies in reproducing in vivo optimal parameters, these could be resolved in the majority of the cases after adjusting some of the initial computed conditions. The latter conditions had to conform to those achieved in the in vivo experiment and the limitations of a simulation approach to a real in vivo situation were then highlighted.

The study focused on spin-echo (FSE and FLAIR) and gradient-echo (GRE and MP-RAGE) based sequences. These methods are commonly used in clinical brain tumor studies and are also relevant to pre-clinical murine brain tumor investigations. The limited inhomogeneity experienced in the spin-echo based methods helped to create a better correlation between the CNR computed and the in vivo values (Fig 2 and S1 Fig). On the other hand, the gradient-echo based sequences being more susceptible to field inhomogeneity effects, produced divergent CNR trends making the pursue of *“optimal in vivo scanning parameters”* a non-trivial task (Fig 3 and S2 Fig). Specifically, the simulation of the standard GRE method revealed to be far from accurate.

FSE was used as a T2- and T1-weighted sequence. As a T2-weighted method, it provided excellent pre-contrast anatomical information and was able to well differentiate normal brain from tumor areas that appeared hyperintense due to increased vascularization. This method is commonly used post-contrast in multiple sclerosis studies, and thus was reported in this work. Post-contrast T2-weighted sequence, however, do not provide any additional biological or tissue structural information compared to a pre-contrast agent administration MRI acquisition. As a T1-weighted method, the post-contrast in vivo data offer important information on the tumor anatomy and permeability effects and therefore is a valuable investigating tool. Simulation and in vivo data were highly correlated as shown by the Kendall's tau coefficient so that the computer program can be used as a reliable surrogate to the in vivo measurements in order to obtain the MRI “*optimal in vivo scanning parameters*”. Of note that particular attention should be given to the ESP scanning parameter because chemical shift effects can represent a limiting factor in achieving well-correlated simulated to in vivo data.

FLAIR is a challenging method when followed by a refocusing flip angle train as studied by Saranathan et al., due to issues related to CSF nulling point signal stability over time, the primary goal of such sequence [28]. Although not often used in pre-clinical studies, FLAIR is routinely used in the clinic making it an essential tool to apply in co-clinical projects. This study shows that in vivo and simulation data well agreed in determining the best TI parameter while having discrepancies in optimizing TR. Such differences could be related to the incrementing number of excitation and refocusing flip angles in the absence of an optimization of the latter refocusing angles. It has to be noted that the number of ETL used at the optimal in vivo TR of 9000 ms was 3 while increasing to 4 at 10000 ms TR. Such increment could lead to variability in signal-to-noise in the acquired images reflecting a drop of CNR between tissues. This method, similarly to the FSET2, is also not commonly used in the post-contrast setting.

Despite being a gradient-echo based method, the 3D MP-RAGE generally reported well-correlated CNR values with high Kendall's tau coefficient between in vivo and simulated approaches. Given that this sequence is most commonly utilized after, rather than prior to contrast agent administration, the simulation can be reliably used to define the “*optimal in vivo scanning parameters*” in post-contrast conditions. Differences between the pre- and post-contrast behavior in identifying the optimal TI value, must lay in a reduction in inhomogeneity effects, which follow decremented T1 values, but also in a better characterization of the tissues. The rather complex equation, presented in the “*Theory*” section, which simulates the MP-RAGE sequence, is therefore offering a good representation of the acquired in vivo signal-to-noise. Of note, contrary to the many parameters which dynamically varied in the simulation, TD was maintained to a “0” value primarily because of a previous publication [22] reporting an enhanced efficacy at such range which was also verified in our laboratory in a separate in vivo experiment (data not reported).

The simulation computer program remains an important tool in MRI parameters optimization and the different C++ codes used in this study are hereby provided and can be freely used (S1, S2, S3, S4 and S5 Files). When engaging such simulations, the user can select and vary the range of several parameters in a dynamic manner in order to automatically achieve the *“optimal scanning computed parameters”* and consequently the “*optimal in vivo scanning parameters*”. As we have seen, however, the computed outcomes do not always accurately represent the in vivo relationship between tissue contrasts. To pursue a simulation which better adheres to in vivo conditions, some of the simulated parameters can no longer change dynamically during the simulation, but must be maintained constant to those found in vivo. Such incoherence between the in vivo and simulation approaches, confirm the latter to be a simplistic method. A numerical calculation does not always take into account in vivo aspects such as field inhomogeneity, hardware limitations and biological effects in addition to artifacts arising from magnetic susceptibility gradients or chemical shift effects that become more pronounced at high static fields [29], thus resulting in misleading interpretations.

Nevertheless, this study proposes some guidelines on how to validate a simulation against an in vivo scenario in order to achieve “*optimal in vivo scanning parameters*”, and highlights the parameters that have to be re-introduced into the numerical simulation after CNR comparison analysis. Our approach is in contrast with the more traditional MRI simulations where a stand-alone computing program uses an analytical approach and theoretical approximation to predict the rather complex magnetization processes of the body under investigation [30–32]. Most of these simulations design elegant, but still job-selective, numerical corrections to overcome either specific hardware limitations, such as RF and magnetic field imperfections, non-linear gradients and gradients field modulation, or image artifacts such as chemical shift, spin dephasing and susceptibility-induced off-resonance. These approaches are generally developed for a clinical environment and/or educational purposes. In contrast, our work, did not aim at resolving specific problematic issues related to signal acquisition, image processing or hardware limitations, but rather to use information gained from in vivo experiments and apply it to some specific scanning parameters to correct the discrepancies seen between the computed and a in vivo scenario, and to forestall the best scanning parameters to use to best differentiate one tissue from the next.

Our proposed simulated approach can be utilized to further improve the accuracy and optimization of scanning parameters by decreasing the range of variation of each variable. For instance, in our spin-echo cases, the TR parameter was varied following an increment of 1000ms, but having proved that such parameter is fully compliant to the simulation, the range of variation could be drastically reduced (100ms for example) and a more accurate value was found from the simulation alone. If the same situation were to be repeated in vivo, the process would consume a considerable amount of time. Higher accuracy of “*optimal scanning in vivo parameters*” will result in further maximized CNRs, and better delineation, differentiation and characterization of the diseased model under study.

The simulation technique reported here is not limited to the glioblastoma murine model but can be used for any type of cancer or tissue. Of note, complex tissue structures, such as necrosis or large edematous regions within a tumor mass, can affect significant areas of the tissue under study, and the T1, T2 or T2* can also significantly vary within the tissue itself. In these cases, the user will have to explore more sophisticated mathematical approaches, rather than an average between relaxation times, to achieve spatial/relaxation “weighted” values in order to reflect the tissue properties, and identify one relaxation time value to use in the simulation. Alternatively, one could test the simulation technique on phantoms with various relaxation times properties before approaching the in vivo experiment. We tested the simulation using a water/oil phantom that provided similar successful and failed scanning parameters to those presented in this work (results not shown). We hope that our proposed methodology can help harmonize the choice of scanning parameters across different scanners, and help validate the use of MRI as an effective tool for translational studies in cancer and other diseases.

In conclusion, this study presented a proposed computer simulation approach for MRI in vivo scanning parameter optimization in a glioblastoma murine model as a validated surrogate for the more time consuming in vivo approach. The programming used to develop this simulation technique relied on a theoretical model and on information provided by the concomitant in vivo study to introduce corrections to the initial simulated conditions as to generate a reliable computed approach and validate the simulation approach against the in vivo acquisition. This proposed method could potentially be extended across pre-clinical and clinical scanners and to other disease models.

## Supporting information

S1 File C++ program file for fast spin-echo. The T1 and T2 values and scanning parameters can be edited within the program file to define pre- and post-contrast conditions.
(CPP)

S2 File C++ program file for FLAIR. The T1 and T2 values and scanning parameters can be edited within the program file to define pre- and post-contrast conditions.
(CPP)

S3 File C++ program fils for gradient-echo. The T1 and T2* and scanning parameters can be edited within the program file to define pre- and post-contrast conditions.
(CPP)

S4 File C++ program file for MPRAGE. The T1 and T2 values and scanning parameters can be edited within the program file to define pre- and post-contrast conditions.
(CPP)

S5 File C++ program file for fast spin-echo with changeable T1 and T2. The T1 T2 and T2 values and scanning parameters can be edited within the program file to define pre- and post-contrast conditions.
(CPP)

S1 Table T1 T2 and T2* variability values for brain parenchyma, CSF and tumor tissues in a glioblastoma murine model for pre-contrast and post-contrast conditions (30 minutes after contrast agent IP injection). (TIFF)

S1 Fig Spin-echo based sequences CNR data. Fast-spin-echo T2- (FSET2) and T1-weighted (FSET1) brain parenchyma-tumor and Fluid Attenuated Inversion Recovery (FLAIR) brain parenchyma-CSF mean CNR graphical representation. In pre-contrast conditions: Figs A_1,3,5_ and A_2,4_,_6_ report in vivo and simulated CNR FSET2 data, respectively, when changing ESP, ETL, TR and NSA (the scan time was limited to 5 minutes). Figs B_1,3_,_5_ and B_2_,_4_,_6_ report in vivo and simulated CNR FSET1 data, respectively, when changing ESP, ETL, TR and NSA (the scan time was limited to 5 minutes). Figs C_1_,_3_ and C_2_,_4_ show in vivo and simulated CNR FLAIR data, respectively, when changing TI, TR and ETL. In this latter case the scan time was limited to 10 minutes. In post-contrast conditions: Figs D_1_,_3_ and D_2_,_4_ reported in vivo and simulated CNR FSET1 data, respectively, when changing ESP, TR and NSA (the scan time was limited to 5 minutes). Black arrows point at selected parameters that typically coincide with the highest CNR providing with the *“optimal in vivo scanning parameters”* in the in vivo approach and the “*optimal computed scanning parameters*” in the simulated approach.
(TIFF)

S2 Fig Gradient-echo based sequences diagrams. Gradient-echo (GRE) and Magnetization-Prepared Rapid Gradient-Echo (MP-RAGE) brain parenchyma-tumor mean CNR graphical representation during scanning parameters optimization. Figs A_1,3_ and A_2,4_ report in vivo and simulated reports in vivo CNR GRE data, respectively, when changing FA and TR in addition to NSA (5-minute scan limit) in pre-contrast conditions. Same approach is shown in Figs C_1_,_3_ and C_2_,_4_ in post-contrast conditions. Figs B_1,3_ and B_2_,_4_ report in vivo and simulated CNR MP-RAGE data, respectively, when changing TI and FA (5-minute scan limit) in pre-contrast conditions. Same approach is shown in Figs D_1_,_3_ and D_2_,_4_ in post-contrast conditions. Black arrows point at selected parameters that typically coincide with the highest CNR providing with the *“optimal in vivo scanning parameters”* in the in vivo approach and the “*optimal computed scanning parameters*” in the simulated approach.
(TIFF)
