# Supplementary figures and images for: Development and validation of a new MRI simulation technique that can reliably estimate optimal in vivo scanning parameters in a glioblastoma murine model
Source: PLoS One. 2018 Jul 23;13(7):e0200611. doi: 10.1371/journal.pone.0200611 (PMC6056046; doi:10.1371/journal.pone.0200611)

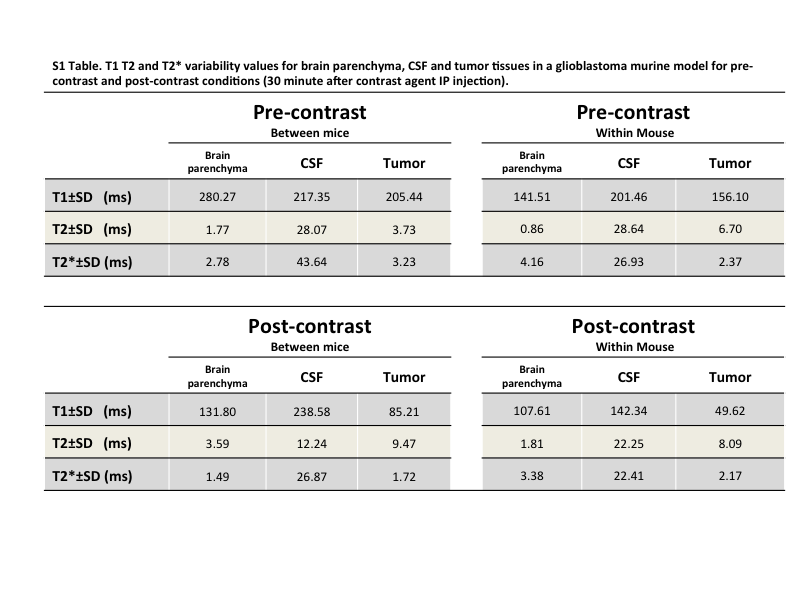

Supplement: S1 Table — (TIFF) [file pone.0200611.s006.tiff]

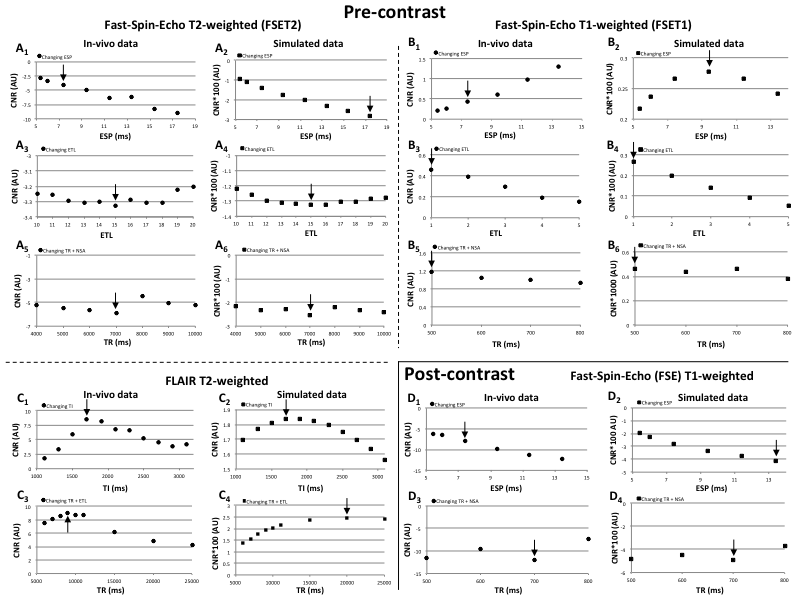

Supplement: S1 Fig — Fast-spin-echo T2- (FSET2) and T1-weighted (FSET1) brain parenchyma-tumor and Fluid Attenuated Inversion Recovery (FLAIR) brain parenchyma-CSF mean CNR graphical representation. In pre-contrast conditions: Figs A1,3,5 and A2,4,6 report in vivo and simulated CNR FSET2 data, respectively, when changing ESP, ETL, TR and NSA (the scan time was limited to 5 minutes). Figs B1,3,5 and B2,4,6 report in vivo and simulated CNR FSET1 data, respectively, when changing ESP, ETL, TR and NSA (the scan time was limited to 5 minutes). Figs C1,3 and C2,4 show in vivo and simulated CNR FLAIR data, respectively, when changing TI, TR and ETL. In this latter case the scan time was limited to 10 minutes. In post-contrast conditions: Figs D1,3 and D2,4 reported in vivo and simulated CNR FSET1 data, respectively, when changing ESP, TR and NSA (the scan time was limited to 5 minutes). Black arrows point at selected parameters that typically coincide with the highest CNR providing with the “optimal in vivo scanning parameters” in the in vivo approach and the “optimal computed scanning parameters” in the simulated approach. (TIFF) [file pone.0200611.s007.tiff]

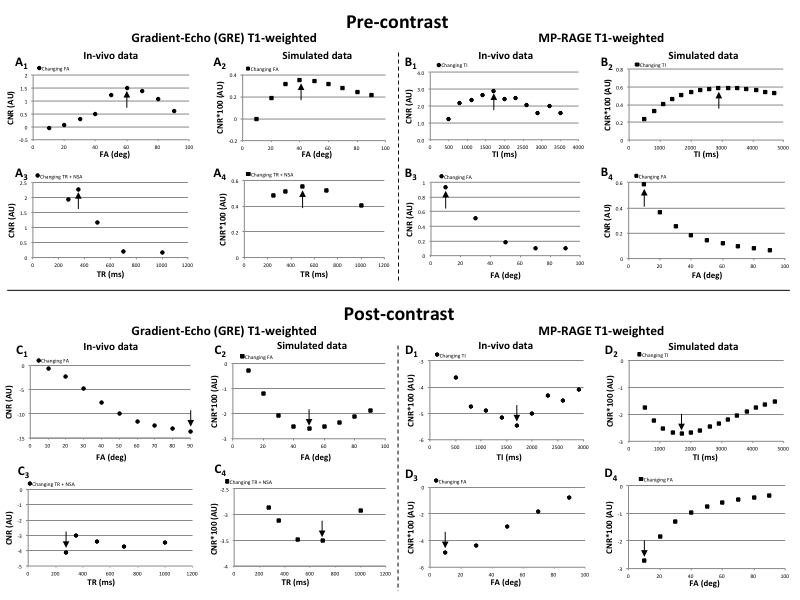

Supplement: S2 Fig — Gradient-echo (GRE) and Magnetization-Prepared Rapid Gradient-Echo (MP-RAGE) brain parenchyma-tumor mean CNR graphical representation during scanning parameters optimization. Figs A1,3 and A2,4 report in vivo and simulated reports in vivo CNR GRE data, respectively, when changing FA and TR in addition to NSA (5-minute scan limit) in pre-contrast conditions. Same approach is shown in Figs C1,3 and C2,4 in post-contrast conditions. Figs B1,3 and B2,4 report in vivo and simulated CNR MP-RAGE data, respectively, when changing TI and FA (5-minute scan limit) in pre-contrast conditions. Same approach is shown in Figs D1,3 and D2,4 in post-contrast conditions. Black arrows point at selected parameters that typically coincide with the highest CNR providing with the “optimal in vivo scanning parameters” in the in vivo approach and the “optimal computed scanning parameters” in the simulated approach. (TIFF) [file pone.0200611.s008.tiff]
